# Impact of portal and systemic venous drainage on early inflammatory responses in a swine model of living donor intestinal transplantation

**DOI:** 10.1016/j.clinsp.2026.100997

**Published:** 2026-05-22

**Authors:** Guilherme F. Paganoti, Uenis Tannuri, Josiane O. Gonçalves, Suellen Serafini, Raimundo R.N. Guimarães, Alessandro R. Belon, Ana Cristina A. Tannuri

**Affiliations:** a Institute for Children and Adolescents at the Universidade de São Paulo, São Paulo, SP, Brazil; b Medical Research Laboratory - Pediatric Surgery (LIM30), São Paulo, SP, Brazil; c Phamarcist in Institute for Children and Adolescents at the Universidade de São Paulo, São Paulo, SP, Brazil; d Medical Research Laboratory in Surgical Technique and Experimental Surgery at Universidade de São Paulo, São Paulo, SP, Brazil

**Keywords:** Intestinal transplantation, Portal venous drainage, Living-donor transplantation, Swine model, Inflammatory response, Interferon-gamma

## Abstract

**Background:**

Venous outflow is a key determinant of immune and metabolic homeostasis following intestinal transplantation. In living-donor settings, maintaining physiologic portal drainage may attenuate systemic inflammation and support local defense mechanisms during early reperfusion. This study investigated the molecular and histological effects of portal versus systemic venous drainage in a swine model of living-donor intestinal transplantation.

**Methods:**

Twenty-two swine intestinal transplants were assigned to portal (n = 12) or caval (n = 10) drainage. Grafts were preserved in Custodiol® (HTK), implanted orthotopically, and sampled two hours after reperfusion. Intestinal and hepatic tissues underwent qRT-PCR analysis of inflammatory, apoptotic, endothelial, and oxidative-stress pathways. Histological injury was scored (0–3). Groups were compared using the Mann-Whitney *U* test with Bonferroni and FDR adjustment.

**Results:**

Histopathological alterations were mild and comparable between groups in both intestinal and hepatic tissues, with no significant differences across epithelial, vascular, inflammatory, or necrotic parameters. In intestinal samples, IL-1α (fold-change = 6.76; p = 0.013) and IFN-γ (fold-change = 2.10; p = 0.023) exhibited higher early expression in portal-drainage grafts, although neither signal remained statistically significant after multiple-testing correction. The remaining markers showed broadly overlapping distributions. In the liver, CD8 demonstrated the largest numerical difference (fold-change = 0.48; p = 0.044), but no hepatic target reached significance after adjustment.

**Conclusions:**

Portal and caval drainage produced comparable early histological injury and only subtle molecular variation. Although not significant after adjustment, these early transcriptional shifts suggest potential immunometabolic effects of venous outflow configuration and represent exploratory, hypothesis-generating findings that warrant further study.

## Highlights

• Portal drainage modulates early intestinal inflammatory gene expression.
• Swine living-donor intestinal transplantation experimental model.
• Portal and caval drainage showed similar early histological injury.
• Early molecular shifts suggest immunometabolic modulation.

## Introduction

Intestinal Transplantation (ITx) remains the most immunogenic of all solid organ transplants, with early graft dysfunction largely driven by ischemia-reperfusion injury and uncontrolled inflammation.^1^ The intestinal mucosa contains an extensive immune network continuously exposed to microbial and dietary antigens, making it highly reactive after reperfusion.^2^ Among the physiological determinants of graft homeostasis, the route of venous drainage plays a critical role. Portal venous outflow, by preserving the enterohepatic circulation, allows the liver to function as an immunometabolic filter, clearing endotoxins, cytokines, and bacterial products before they reach the systemic circulation.^3^, ^4^, ^5^ In contrast, systemic (caval) drainage bypasses hepatic modulation and may amplify systemic inflammation, microvascular stress, and hepatocellular injury. However, the molecular mechanisms linking drainage pattern and early inflammatory signaling remain poorly defined.

Cytokines such as Interferon-Gamma (IFN-γ) and Interleukin-1 alpha (IL-1α) are key mediators at the intestinal interface, exerting dual and context-dependent roles that balance inflammation, defense, and repair. IFN-γ, produced by activated T-cells, NK cells, and innate lymphoid populations, enhances epithelial resistance, antimicrobial peptide expression, and microvascular protection.^5^^,^^6^ Experimental models have shown that local IFN-γ expression within transplanted tissues prevents ischemic necrosis by stabilizing endothelial integrity and inducing Major Histocompatibility Complex (MHC) expression, whereas its absence leads to diffuse parenchymal necrosis despite preserved blood flow, underscoring its cytoprotective and vasoregulatory roles.^6^^,^^7^ IL-1α, in turn, acts as an early alarmin released by stressed enterocytes and endothelial cells, initiating localized repair and controlled immune recruitment while maintaining epithelial barrier integrity.^8^ The coordinated upregulation of IFN-γ and IL-1α represents an adaptive, self-limited inflammatory program that favors graft conditioning and immune tolerance, whereas dysregulation of these pathways promotes barrier breakdown, microvascular failure, and early rejection.^2^^,^^5^

Early inflammatory events following intestinal transplantation are critical determinants of graft performance and host-graft interaction, particularly during the immediate reperfusion phase when endothelial activation, cytokine release, and epithelial stress responses predominate. Within this context, the venous outflow route has been proposed as a modulator of intestinal and hepatic physiology, influencing hemodynamics, mucosal integrity, and the magnitude of ischemia-reperfusion injury. Building upon the authors’ prior experimental work demonstrating that preservation of portal venous drainage may support hepato-intestinal homeostasis and attenuate early tissue injury in a swine model, the authors hypothesized that maintaining physiological portal outflow could influence acute transcriptional responses to reperfusion.^9^ To investigate this possibility, the authors employed a rigorously standardized swine model of living-donor intestinal transplantation and compared early (2-h) gene-expression patterns in grafts reconstructed with portal (mesenteric-mesenteric) versus systemic (mesenteric-caval) venous drainage. This study was intentionally designed as an exploratory, hypothesis-generating analysis aimed at delineating immediate cytokine signatures, rather than evaluating infection-related or functional outcomes, thereby providing mechanistic insights that may inform subsequent, more comprehensive translational studies.

## Materials and methods

### Ethical standards

All animals were handled in accordance with the standards for the didactic-scientific practice of Brazilian vivisection, Law nº11,794, of October 8, 2008, Decree nº6,899, of July 15, 2009, which regulates procedures with animals submitted to scientific research, and resolution nº714 of June 20, 2002, of the Federal Council of Veterinary Medicine (CFMV), which regulates euthanasia procedures. The Ethics Committee for the Use of Animals (CEUA ‒ Faculty of Medicine of USP ‒ SP) approved this project with Protocol number 1355/2019.

This study was conducted and reported in accordance with the ARRIVE 2.0 guidelines for animal research.

### Experimental design

A total of 22 Living-Donor Intestinal Transplantations (LDITs) were successfully completed in Large White pigs and allocated to either the Portal group (n = 12) or the Caval group (n = 10). The minor imbalance in sample size resulted from two intraoperative exclusions in the systemic drainage arm, both unrelated to the experimental hypothesis: one due to hemodynamic instability during anesthesia induction and another due to technical failure of vascular anastomosis. Only animals that fulfilled all predefined criteria for procedural completion were included in the final analysis. Donor pigs weighed 33–70 kg, and recipients weighed 18–28 kg.

### Anesthesia and monitoring

Thirty minutes before induction, both donors and recipients received intramuscular xylazine (2 mg/kg) and ketamine (10 mg/kg) as pre-anesthetic medication. Anesthesia was induced with propofol (3–5 mg/kg) and maintained with isoflurane (1.5%–2.5%) in oxygen and fentanyl (0.1 μg/kg/min) by continuous infusion. Mechanical ventilation was performed using an InterMed® Interlinea A anesthesia machine. Invasive monitoring included continuous ECG, pulse oximetry, capnography, body temperature, invasive arterial pressure, and central venous pressure. A 7 French (Fr) double-lumen jugular catheter was inserted for fluid infusion and venous sampling, and a femoral arterial catheter was placed for blood pressure and gas analysis.

### Donor surgery

All donor operations were performed by the same surgeon using 3.5× magnification loupes and an auxiliary light source. Ampicillin (50 mg/kg) and cefotaxime (50 mg/kg) were administered before laparotomy. A midline incision from the xiphoid to the pubis was performed, and exposure was maintained with a Gosset retractor. After evisceration, an ileal segment corresponding to 20% of the total intestinal length was selected retrogradely, preserving the distal 20 cm near the ileocecal valve. The mesentery was carefully dissected, isolating the corresponding arterial and venous branches of the superior mesenteric vessels to obtain a 2–3 cm vascular pedicle. Resection was performed within the defined ischemic boundaries using a linear stapler, and vascular ligations were secured with 4‒0 Prolene (Ethicon) sutures. In Caval group donors, a segment of the inferior vena cava between the renal and iliac veins was harvested for later use as an interposition graft. Additionally, approximately 300 mL of autologous blood was collected from the aorta via a 14Fr catheter and transfused into the recipient at reperfusion. After procurement, the donor was euthanized by exsanguination, and the abdominal wall was closed with a continuous 3‒0 nylon (Ethicon) suture.

### Back-table procedure

The intestinal graft was transferred to a sterile back-table setup and maintained on ice. The artery was cannulated, and the graft was perfused sequentially with 1 L of cold 0.9% saline containing 5 mL of heparin (5000 IU/mL), followed by 1 L of Custodiol® (HTK, Bensheim, Germany) preservation solution to ensure complete vascular flushing. A final rinse was performed with 250 mL of saline mixed with 50 mL of 20% human albumin to stabilize the microvascular endothelium. The graft was kept immersed in cold preservation solution throughout the procedure, and vascular ends were prepared for implantation. No luminal cleansing or bowel decontamination was performed at any stage.

### Recipient surgery

Recipient operations were performed under identical anesthetic and monitoring protocols as the donors. After a midline xiphoid-pubic laparotomy and full evisceration, the superior mesenteric artery and vein were ligated at their origins, and the entire small intestine was resected, preserving the duodenum and rectum to simulate a short-bowel condition.

In the Portal group, venous outflow from the graft was reconstructed by end-to-end anastomosis between the graft and recipient superior mesenteric veins, restoring physiologic portal drainage. In the Caval group, the graft vein was anastomosed end-to-side to the inferior vena cava, approximately 2.5 cm above the right renal vein, ensuring a straight trajectory and avoiding angulation of the interposed venous graft. Arterial inflow was established by end-to-end anastomosis between the graft and the recipient superior mesenteric arteries.

Intestinal continuity was restored through double-layer end-to-end anastomoses between the graft and recipient duodenum (proximal) and rectum (distal) using 5‒0 Prolene sutures (Ethicon). Before vascular unclamping, methylprednisolone (20 mg/kg) was administered intravenously. Immediately after reperfusion, bicarbonate (5 mEq/10 kg) and calcium chloride (10 mg/kg) were infused to correct acidosis and stabilize myocardial membranes.

After two hours of reperfusion, liver and intestinal biopsies were obtained for molecular studies.

### Tissue sampling and molecular analysis

All histopathological evaluations were performed by an experienced pathologist who was fully blinded to group allocation and experimental conditions. Semi-quantitative scoring was conducted using standardized reference criteria derived from healthy, freshly anesthetized swine without surgical manipulation, providing a physiological baseline for comparison of intestinal and hepatic architecture. Tissue sections (4 µm) were stained with Hematoxylin-Eosin (HE) and examined under light microscopy.

Intestinal samples were assessed for edema, vascular congestion, lymphocyte infiltration, epithelial desquamation, villous and crypt architectural distortion, and mucosal apoptosis. Hepatic samples were evaluated for inflammatory infiltrate, hepatocellular necrosis, or parenchymal edema. Each parameter was graded using a semi-quantitative scale ranging from 0 (absent) to 3 (severe). This blinded and standardized approach ensured a consistent, objective assessment of tissue injury across experimental groups.

Fragments of the intestinal graft and liver were collected from the transplanted graft two hours after reperfusion. Additionally, liver and intestinal tissue from recipient animals at time zero (immediately after laparotomy and before any surgical manipulation, ischemia, or reperfusion) were collected exclusively to generate a pooled calibrator sample for relative gene expression analysis using the 2^^-ΔΔCt^ method, and were not included in the experimental groups. All tissue samples were immediately frozen in liquid nitrogen and stored at −80 °C until further processing.

Total RNA was extracted from approximately 10 mg of tissue using TRIzol® reagent (Invitrogen, USA), following homogenization in a Mikro-Dismembrator U (Sartorius, Germany). RNA purity was assessed by spectrophotometry (A260/A280 ratios between 1.8 and 2.0), and RNA integrity was evaluated by 1.2% agarose gel electrophoresis, confirming intact 18S and 28S rRNA bands.

Complementary DNA (cDNA) was synthesized from 4 µg of total RNA using SuperScript III Reverse Transcriptase (Invitrogen, USA) and Oligo(dT) primers, following the manufacturer’s protocol. For pooled control samples used as calibrators in relative quantification, RNA from eight healthy animals was individually extracted, quantified, and combined in equimolar amounts to generate a single pooled RNA sample, from which 4 µg was used for cDNA synthesis. This pooled sample represents the basal physiological state of intestinal and hepatic tissue, minimizing inter-individual variability. qRT-PCR was performed on a Rotor-Gene *Q* 5-plex HRM thermal cycler (Qiagen, Germany) using SYBR Green I detection (Invitrogen, USA). Each 15 µL reaction contained 100 ng of cDNA, 0.3 µL of gene-specific forward and reverse primers (10 µM each), and 7.5 µL of Platinum SYBR Green qRT-PCR SuperMix-UDG (Invitrogen, USA). Reactions were performed in technical triplicates, and negative controls were included to monitor potential contamination. The cycling protocol consisted of an initial denaturation at 95 °C for 5 min, followed by 40 cycles of 95 °C for 20 s, 60 °C for 30 s, and 72 °C for 30 s. A final melting-curve analysis was performed to confirm amplification specificity and the absence of primer-dimer artifacts. Primer sequences, amplicon sizes, and in silico validation against the Sus scrofa genome are provided in Supplementary Table 1. Melting curve analyses demonstrating single-peak amplification for all target genes are shown in Supplementary Fig. 1, while amplification efficiency, linear dynamic range and correlation coefficients (R²) are reported in Supplementary Table 2.

Gene expression was assessed for BAX, BCL-XL, IL-1α, IL-1β, IL-6, IL-17, TNF-α, IFN-γ, iNOS, eNOS, CXCL10, and CD8. Candidate reference genes, including β-actin, HPRT, B2M, GAPDH, and YWHAZ, were evaluated for expression stability across all experimental groups using the online tool RefFinder (https://www.ciidirsinaloa.com.mx/RefFinder-master), which integrates geNorm, NormFinder, BestKeeper, and comparative ΔCt algorithms. A comprehensive ranking of stability scores was generated for each tissue type (liver and intestine), and HPRT was selected as the most stable reference gene for both tissues (Supplementary Tables 3 and 4). Relative gene expression was calculated by the Livak (2^^-ΔΔCt^) method, with the pooled healthy control samples serving as the calibrator. Fold changes are expressed relative to this baseline.

### Statistical analysis

All statistical analyses were performed using Python (NumPy, SciPy, Pandas, and Matplotlib). Continuous variables are presented as medians due to the non-normal distribution of molecular expression values. For each of the 12 molecular markers analyzed in the intestine and liver (24 total comparisons), the authors compared groups using the two-tailed Mann-Whitney *U test*. To address the multiplicity of testing, the authors applied both Bonferroni correction (αadjusted = 0.05/24 = 0.0021) and Benjamini-Hochberg False-Discovery-Rate (FDR) adjustment, reporting raw p-values, Bonferroni-adjusted p-values, and q-values for all markers. Effect sizes were quantified as the fold-change between median expression in the portal and caval groups. To provide distribution-free confidence intervals, fold-changes were bootstrapped with 5000 resamples, generating 95% bias-corrected accelerated confidence intervals for each marker.

## Results

### Operative and morphometric parameters

A total of 22 living-donor intestinal transplantations were performed, divided into portal (n = 12) and caval (n = 10) venous drainage groups. Donor weight ranged from 33 to 70 kg, while recipient weight varied between 18 and 28 kg. The median cold ischemia time was 153 min (IQR 130–180) in the Portal group and 175 min (IQR 160–190) in the Caval group. Warm ischemia times were 31.5 min (IQR 27–34) and 33 min (IQR 30–39), respectively. Median graft length corresponded to 20% of the total small bowel, measuring 500 cm (IQR 400–500) in the Portal group and 500 cm (IQR 500–500) in the Caval group. Donor and recipient body weights were comparable between groups, as were the intraoperative ischemia times. Descriptive data and Mann-Whitney *U* test results are summarized in Table 1.

**Table 1:** Morphometric and operative parameters.

**Variable**	**Portal Median (IQR)**	**Caval Median (IQR)**	**p-value**
	**n=12**	**n=10**	
Donor weight (kg)	42.5 (38.0–46.0)	40.0 (36.0–67.0)	0.716
Recipient weight (kg)	22.0 (20.0–23.0)	21.6 (20.0–24.0)	0.791
Cold ischemia time (min)	153.0 (130.0–180.0)	175.0 (160.0–190.0)	0.129
Warm ischemia time (min)	31.5 (27.0–34.0)	33.0 (30.0–39.0)	0.425
Graft size (cm)	500 (400–500)	500 (500–500)	0.027

Mann-Whitney *U*.

### Intestinal pathology

Histopathological evaluation of intestinal grafts demonstrated predominantly mild to moderate alterations across both experimental groups. Semi-quantitative scoring revealed low overall frequencies of epithelial denudation, villous blunting, and vascular congestion in both drainage configurations, with most samples scoring between 0 and 2 on the 0–3 scale. Apoptosis of mucosal epithelium and crypt reactivity were rare findings, observed in only a small subset of animals. Lymphocytic epithelial permeation and vascular inflammation were also infrequent and remained within the lower severity categories. No statistically significant differences were detected between portal and caval drainage for any of the evaluated parameters, indicating that early (two-hour) post-reperfusion histological changes were subtle and largely comparable across groups. Within the immediate reperfusion window, structural injury is limited and does not diverge meaningfully according to venous outflow route. Descriptive data and Mann-Whitney *U test* results are summarized in Table 2.

**Table 2:** Intestinal pathological feature.

**Variable**	**Portal Median (IQR)**	**Caval Median (IQR)**	**p-value**
	**n=12**	**n=10**	
Epithelial Denudation	0.5 (0.0–2.0)	0.5 (0.0–1.0)	0.749
Mucosal Apoptosis	0.0 (0.0–0.0)	0.0 (0.0–0.0)	0.411
Villous Blunting	0.0 (0.0–1.2)	0.0 (0.0–1.0)	0.908
Lymphocytic Epithelial Infiltration	1.0 (1.0–2.0)	1.0 (1.0–1.8)	0.643
Vascular Congestion	0.0 (0.0–1.0)	0.5 (0.0–1.0)	0.739

Semi-quantitative hepatic histopathological findings comparing portal and caval venous drainage groups two hours after reperfusion. Values are presented as median (interquartile range). Scoring criteria ranged from 0 = Absent, 1 = Mild, 2 = Moderate, to 3 = Severe. Data are presented as median (IQR); comparisons between groups were performed using the Mann-Whitney *U test*.

### Intestinal gene expression

**Fig. 1:**
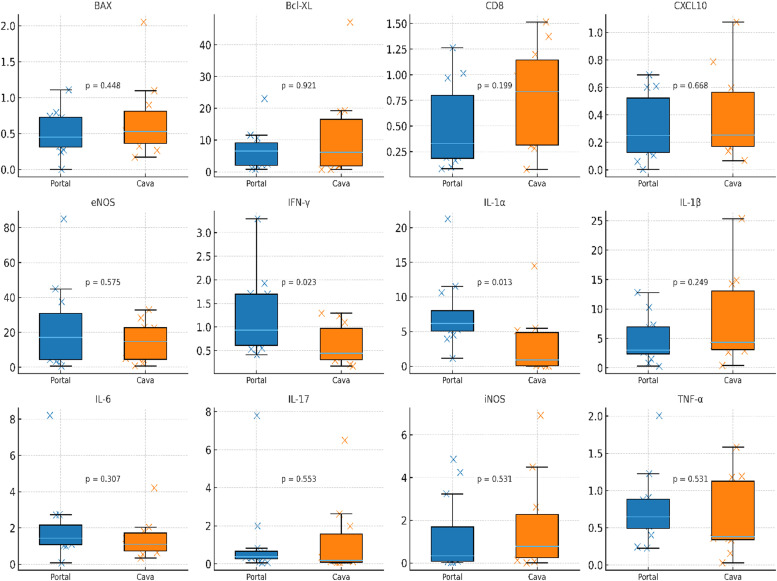
Distribution of intestinal molecular marker expression in portal versus systemic venous drainage groups after 2-h reperfusion. Intestinal mRNA expression of inflammatory, apoptotic, and endothelial markers comparing Portal (blue) and Caval (orange) venous drainage. Boxplots show median and interquartile range, with all individual values overlaid; p-values indicate Mann-Whitney *U test* results.

Early post-reperfusion analysis of intestinal tissue revealed distinct quantitative patterns across the assessed molecular markers. Median IL-1α expression was higher in the portal group (6.18) compared with the caval group (0.91), with a corresponding fold-change of 6.76 (95% CI 1.15–31.11). IFN-γ expression also differed, with median values of 0.94 in portal animals and 0.45 in caval animals, yielding a fold-change of 2.10 (95% CI 0.80–3.48). For the remaining markers ‒ including BAX, BCL-XL, CD8, CXCL10, eNOS, IL-1β, IL-6, IL-17, iNOS, and TNF-α ‒ median values were broadly overlapping between groups, and bootstrap-derived fold-change confidence intervals included 1.00. Mann–Whitney *U* testing produced unadjusted p-values of 0.013 for IL-1α and 0.023 for IFN-γ, while all other comparisons yielded p > 0.19. After multiple-testing correction (Bonferroni and Benjamini-Hochberg FDR), no intestinal marker met the threshold for statistical significance (Table 3 and Fig. 1).

**Table 3:** Intestinal gene expression according to venous drainage pattern two hours after reperfusion.

**Marker**	**Median Portal**	**Median Caval**	**FoldChange P/C**	**FC CI-low**	**FC CI-high**	**p-value**	**p-Bonferroni**	**q-FDR**
BAX	0.45	0.531	0.848	0.46	1.519	0.448	1	0.69
BclXL	6.536	6.138	1.065	0.19	4.603	0.921	1	0.921
CD8	0.332	0.835	0.397	0.181	2.101	0.199	1	0.476
CXCL10	0.251	0.254	0.988	0.308	2.4	0.668	1	0.764
eNOS	17.138	14.836	1.155	0.235	5.791	0.575	1	0.69
IFNg	0.936	0.446	2.095	0.799	4.82	0.023	0.55	0.275
IL1a	6.178	0.914	6.756	1.145	195.257	0.013	0.322	0.275
IL1b	3.02	4.338	0.696	0.203	1.76	0.249	1	0.542
IL6	1.44	1.112	1.295	0.729	2.442	0.307	1	0.553
IL17	0.376	0.182	2.058	0.175	5.81	0.553	1	0.69
iNOS	0.334	0.777	0.43	0.056	4.71	0.531	1	0.69
TNFa	0.646	0.38	1.702	0.503	2.596	0.531	1	0.69

Values are expressed as medians. Fold-change was calculated as the ratio of median expression in the Portal group over the Caval group (P/C). Confidence intervals for fold-change were estimated by bootstrap resampling (5000 iterations). p-values refer to Mann-Whitney *U tests* comparing Portal versus Caval drainage. Bonferroni-adjusted p-values (p-Bonferroni) and Benjamini-Hochberg false discovery rate-adjusted q-values (q-FDR) were calculated across all 24 molecular comparisons (12 intestinal and 12 hepatic markers).

### Hepatic pathology

Hepatic histopathological evaluation revealed generally low-grade alterations in both groups. Portal inflammation ranged from 0 to 1 across specimens and showed similar distributions between portal and caval drainage. Lobular inflammation was absent in most samples, with occasional mild foci observed in both groups. Necrosis was uncommon and limited to focal, low-intensity findings. Ballooning degeneration exhibited greater variability, particularly in the caval group, but remained absent or mild in most animals. Fibrosis was not detected in any specimen. Vascular congestion demonstrated a wider distribution, with scores ranging from 0 to 3, and was observed more frequently among portal-drainage grafts. Full quantitative results, including median scores, interquartile ranges, and statistical comparisons, are summarized in Table 4.

**Table 4:** Liver pathological feature.

**Variable**	**Portal Median (IQR)**	**Caval Median (IQR)**	**p-value**
	**n=12**	**n=10**	
Portal Inflammation	0.0 (0.0–1.0)	0.0 (0.0–1.0)	0.782
Necrosis	0.0 (0.0–0.0)	0.0 (0.0–0.8)	0.218
Fibrosis	0.0 (0.0–0.0)	0.0 (0.0–0.0)	1
Lobular Inflammation	0.0 (0.0–0.0)	0.0 (0.0–0.8)	0.218
Ballooning	0.0 (0.0–0.2)	1.5 (0.0–2.0)	0.126
Congestion	1.0 (0.0–1.0)	0.0 (0.0–0.0)	0.029

Semi-quantitative hepatic histopathological findings comparing portal and caval venous drainage groups two hours after reperfusion. Values are presented as median (interquartile range). Scoring criteria ranged from 0 = Absent, 1 = Mild, 2 = Moderate, to 3 = Severe; p-values were obtained using the two-tailed Mann-Whitney *U test*.

### Hepatic gene expression

**Fig. 2:**
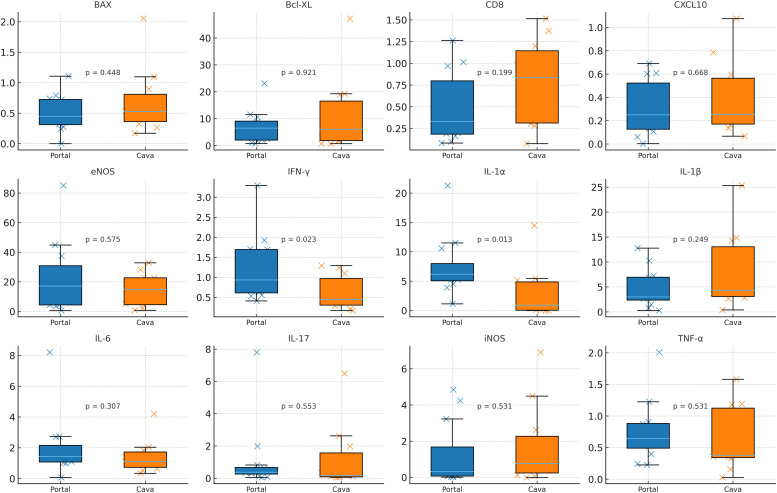
Differential hepatic gene expression two hours after reperfusion in portal and caval drainage models. Hepatic mRNA expression of inflammatory, apoptotic, and endothelial markers comparing Portal (blue) and Caval (orange) drainage groups. Boxplots show median/IQR with all individual values overlaid; p-values reflect Mann-Whitney *U tests*.

In hepatic samples collected two hours after reperfusion, CD8 showed the largest numerical difference between groups, with median expression of 1.18 in the portal group versus 2.46 in the caval group (fold-change = 0.48; 95% CI 0.21–1.43). Other markers, including BAX, BCL-XL, CXCL10, eNOS, IFN-γ, IL-1α, IL-1β, IL-6, IL-17, iNOS, and TNF-α, demonstrated comparable median values between groups, and all fold-change confidence intervals encompassed 1.00. The unadjusted Mann-Whitney *U* p-value for CD8 was 0.044, whereas all other hepatic markers showed p > 0.06. Following Bonferroni and Benjamini-Hochberg adjustments, none of the hepatic comparisons remained statistically significant (Table 5 and Fig. 2).

**Table 5:** Hepatic gene expression according to venous drainage pattern two hours after reperfusion.

**Marker**	**Median Portal**	**Median Caval**	**FoldChange P/C**	**FC CI-low**	**FC CI-high**	**p-value**	**p Bonferroni**	**q-FDR**
BAX	0.059	0.044	1.326	0.61	2.641	0.322	1	0.553
BclXL	0.022	0.072	0.306	0.117	1.057	0.06	1	0.289
CD8	1.018	2.371	0.429	0.184	1.427	0.044	1	0.289
CXCL10	0.058	0.08	0.727	0.1	1.8	0.531	1	0.69
eNOS	0.058	0.114	0.507	0.142	1.352	0.106	1	0.425
IFNg	0.005	0.007	0.769	0.157	1.834	0.274	1	0.548
IL1a	0.012	0.02	0.641	0.075	5.733	0.766	1	0.836
IL1b	0.01	0.038	0.276	0.057	1.238	0.06	1	0.289
IL6	0.041	0.098	0.421	0.106	1.903	0.187	1	0.476
IL17	0.506	1.014	0.499	0.09	2.172	0.176	1	0.476
iNOS	0.338	0.274	1.235	0.283	3.52	0.921	1	0.921
TNFa	0.021	0.026	0.808	0.204	1.719	0.176	1	0.476

Values are expressed as medians. Fold-change was calculated as the ratio of median expression in the Portal group over the Caval group (P/C). Confidence intervals for fold-change were estimated by bootstrap resampling (5000 iterations). p-values refer to Mann-Whitney *U tests* comparing Portal versus Caval drainage. Bonferroni-adjusted p-values (p-Bonferroni) and Benjamini-Hochberg false discovery rate-adjusted q-values (q-FDR) were calculated across all 24 molecular comparisons (12 intestinal and 12 hepatic markers).

## Discussion

This study was conceived as an exploratory, hypothesis-generating investigation aimed at characterizing early cytokine and immunometabolic patterns following intestinal transplantation with portal versus systemic venous drainage, rather than assessing downstream clinical outcomes. As expected in a swine model with inherently limited sample size, statistical power was modest, and only a few markers reached nominal significance, none of which persisted after conservative correction for multiple testing. Importantly, baseline demographic and perioperative variables did not differ between groups, reducing the likelihood of confounding and supporting the interpretation that any early molecular divergence is more plausibly related to the venous drainage configuration itself. These considerations mandate cautious interpretation, and the transcriptional shifts observed here should be viewed as preliminary biological signals rather than conclusive mechanistic evidence. Even so, the delineation of these early expression patterns establishes a physiological framework that can guide larger studies employing extended timepoints, mechanistic assays, and integrated functional endpoints.

Intestinal transplant recipients experience the highest incidence of infectious complications among all types of abdominal organ transplants, since the graft is heavily colonized by microorganisms and exhibits intense immune activity. Current clinical evidence emphasizes that systemic sepsis, infections caused by multidrug-resistant bacteria, and intra-abdominal abscesses remain the leading causes of morbidity and mortality in this setting, despite major advances in antimicrobial therapy and postoperative care.^10^^,^^11^ Within this high-risk context, comparative studies in humans have demonstrated that routing mesenteric venous outflow through the liver markedly reduces the occurrence of infections caused by enteric bacteria.^3^ In a cohort directly comparing portal versus systemic venous drainage in small-bowel transplantation, the cumulative incidence of bloodstream or respiratory infections by intestinal microorganisms (mainly *Enterobacteriaceae* and *Enterococcus* species) was significantly lower among recipients with portal drainage, consistent with a hepatic first-pass clearance effect.^3^^,^^4^ Furthermore, the same study described a pharmacological first-pass phenomenon, evidenced by higher tacrolimus requirements in recipients with portal drainage, supporting the notion that the liver effectively filters and metabolically conditions mesenteric blood before it reaches the systemic circulation.^3^

These clinical findings are reinforced by prior evidence demonstrating that bacterial translocation from the intestinal lumen to the bloodstream is a major mechanism underlying early post-transplant infections.^10^^,^^11^ Additionally, pediatric series have shown that invasive fungal infections ‒ particularly intra-abdominal candidiasis ‒ tend to cluster in the early postoperative period, reflecting the graft’s immunological vulnerability.^11^

The current experimental data are consistent with these observations. In a swine model of living-donor intestinal transplantation, portal venous drainage was associated with early upregulation of Interleukin-1 alpha (IL-1α) within the intestinal graft, indicating an activated but regulated immune profile.^9^ These cytokines play essential roles in reinforcing epithelial integrity and coordinating innate immune defense, suggesting that portal drainage may favor a physiological immune equilibrium that enhances local microbial control while preventing systemic dissemination of pathogens.^9^ Together, these clinical and experimental data support the concept that physiological portal outflow contributes to improved infectious outcomes after intestinal transplantation through both hepatic clearance mechanisms and controlled mucosal immune activation.

The higher intestinal IFN-γ expression observed in grafts with portal drainage at two hours post-reperfusion may be consistent with early antimicrobial signaling, although this isolated transcriptional change requires cautious interpretation. In human intestinal epithelial cell models, IFN-γ directly reduces enterocyte infection by *Cryptosporidium parvum* through mechanisms involving inhibition of pathogen invasion and modulation of intracellular iron metabolism. These effects are mediated by JAK/STAT-dependent and iNOS-independent pathways, demonstrating an autonomous epithelial response that enhances barrier resistance against intracellular pathogens.^7^

More broadly, Interferon-gamma (IFN-γ) functions as a central orchestrator of antimicrobial immunity, inducing a transcriptional program that integrates epithelial and myeloid defenses.^12^ Among its key effector pathways are the PKR/eIF-2α system, transiently suppressing protein synthesis to limit intracellular pathogen replication, and the OAS/RNase L system, which promotes the degradation of microbial and host RNA as part of a controlled antiviral and homeostatic response.^12^ In parallel, IFN-γ enhances antigen presentation through upregulation of both class I and class II Major Histocompatibility Complex (MHC) molecules and can induce Nitric Oxide Synthase 2 (NOS2/iNOS) expression in appropriate contexts. Collectively, these mechanisms underscore the role of IFN-γ as a pivotal link between epithelial resilience and immune activation within the intestinal mucosa.^12^

In phagocytic cells, the antimicrobial “imprinting” induced by Interferon-gamma (IFN-γ) extends to the control of intracellular pathogens through Nitric Oxide Synthase-2 (NOS2) ‒ mediated mechanisms. In murine macrophages, IFN-γ priming upregulates a network of restriction factors ‒ including NOS2 ‒ that collectively enhance the cell’s capacity to restrain intracellular organisms.^13^ This response is partially modulated by innate sensing pathways involving Interferon Regulatory Factor-3 (IRF3), yet the induction of NOS2 remains a hallmark of the IFN-γ ‒ activated macrophage phenotype.^13^ These findings highlight the mechanistic link between IFN-γ signaling and the early containment of microbial dissemination, potentially relevant to the improved systemic and portal immune control observed after graft implantation.

In the context of intestinal transplantation, experimental studies have demonstrated that the Interferon-gamma (IFN-γ) axis contributes to the dynamics of mucosal injury and rejection ‒ a role that, while not obligatory, remains biologically relevant and depends on the expression of IFN-γ receptors in the graft epithelium.^14^ This dual function highlights the bifaceted nature of IFN-γ, acting both as an antimicrobial defense mediator and as a modulator of alloimmune responses. Consequently, an early and compartmentalized elevation of IFN-γ, as observed in the portal drainage group, may represent a localized protective response that enhances microbial containment without necessarily promoting excessive tissue injury when properly regulated.

Thereby, current literature supports that an early post-reperfusion surge of Interferon-gamma (IFN-γ) within the intestinal epithelium promotes direct cellular resistance and primes innate immune circuits for pathogen restriction.^14^ These mechanisms are consistent with the present finding of elevated IFN-γ expression in the intestinal grafts with portal venous drainage, suggesting that early, localized activation of IFN-γ may contribute to improved microbial control and graft stabilization during the immediate post-transplant period.

The selective postoperative upregulation of Interleukin-1α (IL-1α) observed in the intestinal tissue of the portal drainage group is consistent with the well-recognized role of IL-1α as a constitutive epithelial “alarmin”, mobilized upon intestinal surgeries or mechanical stress.^15^ IL-1α is present in an active precursor form in intestinal epithelial and endothelial cells, acting as an immediate sentinel of damage.^16^ Upon release, it triggers autocrine and paracrine reinforcement of junctional integrity through induction of adhesion molecules and tight-junction proteins, thereby limiting paracellular permeability and bacterial translocation.^16^^,^^17^ Experimental studies have shown that exogenous IL-1α administration mitigates mucosal permeability and reduces bacterial translocation after intestinal ischemia-reperfusion and burn injury, emphasizing its barrier-protective and antimicrobial properties.^17^^,^^18^

At the mucosal interface, IL-1α serves as an upstream coordinator of innate immunity, recruiting neutrophils and macrophages for rapid microbial clearance.^19^ In models of enteric infection, IL-1α deficiency impairs early chemokine induction and delays neutrophil migration, resulting in higher pathogen loads.^15^ This early epithelial signaling loop aligns with the physiological advantage conferred by portal drainage, which maintains hepatosplanchnic circulation and may enhance clearance of inflammatory mediators reaching the liver. Within this framework, the elevated IL-1α expression in portal-drained grafts likely reflects a regulated epithelial activation ‒ sufficient to strengthen the barrier and promote innate recruitment without provoking excessive or systemic inflammation.

Thus, integrating the experimental data with current evidence suggests that portal venous drainage may foster an IL-1α-driven immunometabolic adaptation, characterized by localized epithelial defense, efficient microbial containment, and coordinated resolution of early reperfusion stress.^9^^,^^15^, ^16^, ^17^, ^18^, ^19^ This mechanistic alignment reinforces the hypothesis that the IL-1α induction observed during portal drainage may contribute to a transient, yet protective, mucosal immune activation, favoring better infection control in the immediate post-transplant period.

The higher concentration of hepatic CD8⁺ mRNA observed in animals with systemic venous drainage can be interpreted in light of the complex immunologic dynamics that follow intestinal transplantation. Experimental and clinical studies have shown that intestinal transplantation induces persistent activation of the adaptive immune system, characterized by expansion of cytotoxic subpopulations and sustained inflammatory signaling, even in the absence of overt rejection.^20^ This chronic activation reflects continuous systemic exposure to antigens and microbial products derived from the intestinal mucosa ‒ an effect that becomes more pronounced when graft venous return occurs through the systemic route, bypassing the hepatic immunometabolic filter.

In mesenteric-caval drainage, portal blood from the graft reaches the systemic circulation directly, allowing cytokines, bacterial components, and antigenic fragments to rapidly disseminate to secondary lymphoid organs and to the liver through retrograde flow. This nonphysiologic route can elicit a broader and more intense immune response, promoting recruitment of effector CD8⁺ lymphocytes to the hepatic parenchyma. Indeed, experimental models of liver transplantation have demonstrated that the liver, far from being an “immunoprivileged” organ, is capable of priming naïve CD8⁺ T-cells and generating robust cytotoxic responses when exposed to alloantigens under inflammatory conditions.^21^

A more cautious interpretation of the CD8 findings is warranted. Although systemic drainage showed higher CD8 mRNA levels in the liver, this transcriptional signal cannot be directly interpreted as increased lymphocytic infiltration or cytotoxic activity. Consistent with the exploratory nature of the study, the authors refrained from attributing functional meaning to isolated cytokine or CD8 differences, acknowledging that mRNA abundance does not equate to protein expression, immune activation, or histological recruitment in the absence of immunohistochemical or functional validation.

Although intestinal IFN-γ and IL-1α transcripts and hepatic CD8⁺ mRNA levels showed nominal elevations, none of these differences ‒ nor those observed across the remaining markers ‒ reached statistical significance after correction for multiple testing, indicating that the overall transcriptional profiles between drainage configurations remain broadly comparable within the exploratory scope of this study. Expression levels of IL-1β, IL-6, TNF-α, IL-17, iNOS, eNOS, CXCL10, BAX, and BCL-XL remained comparable between groups, suggesting that the early phase assessed ‒ two hours after reperfusion ‒ represents a transitional period dominated by immediate inflammatory and ischemia-reperfusion responses rather than by secondary regulatory cascades. This pattern is consistent with prior experimental models of intestinal transplantation, which demonstrate that most inflammatory and apoptotic transcripts display delayed modulation, typically becoming significant after 2‒6 h post-reperfusion, once transcriptional feedback and cellular recruitment intensify.^22^^,^^23^ Moreover, the variability intrinsic to swine models and the small sample size inherent to survival-limited experimental transplantation may have reduced statistical power to detect subtle differences. Accordingly, the absence of statistically supported divergence across the broader transcriptional panel does not negate potential biological relevance, but rather underscores the selective and preliminary nature of these early IFN-γ^-^ and IL-1α^-^associated signals in the immediate post-reperfusion period.

When the molecular and histopathological datasets are examined together, a coherent pattern emerges. Early reperfusion is characterized by subtle, selective biochemical activation rather than overt tissue damage. Although IL-1α and IFN-γ in the intestine and CD8 in the liver exhibited modest early divergence between portal and caval drainage, these transcriptional shifts did not translate into detectable structural injury. Both intestinal and hepatic specimens showed uniformly mild, nonspecific alterations ‒ such as low-grade epithelial or vascular changes in the intestine and minimal portal or lobular inflammation in the liver ‒ without statistically significant differences between groups. This convergence of findings indicates that transcriptional perturbations during the initial hours of reperfusion likely represent upstream immuno-metabolic signaling events that precede the development of macroscopic lesions. The lack of histopathological separation between drainage strategies, therefore, reflects not an absence of biological effect, but rather the early phase of ischemia-reperfusion dynamics, in which molecular sensitivity exceeds the morphologic threshold for injury. Collectively, these results support an interpretation of limited and targeted early molecular variation, rather than broad inflammatory or cytotoxic divergence, across portal and systemic venous outflow configurations.

## Conclusion

In this exploratory swine model of living-donor intestinal transplantation, portal venous drainage was associated with modest early transcriptional differences, most notably higher intestinal IFN-γ and IL-1α expression and a trend toward lower hepatic CD8 mRNA levels compared with systemic drainage. Although these findings did not persist after conservative correction for multiple testing and should not be interpreted as evidence of functional protection or physiologic superiority, they delineate discrete early molecular signals that warrant further investigation. Rather than supporting definitive mechanistic conclusions, the present results highlight the potential influence of venous outflow configuration on acute post-reperfusion inflammatory patterns and provide a hypothesis-generating framework for future studies incorporating larger cohorts, serial sampling, and integrated histologic, proteomic, and functional endpoints.

## Data availability statement

All data generated or analyzed during this study are included in this published article. Additional information is available from the corresponding author upon reasonable request.

## Funding

Sources of Support: This study was supported by a scholarship from the São Paulo Research Foundation (FAPESP ‒ *Fundação de Amparo à Pesquisa do Estado de São Paulo*). Project Number 2019/23270-0.

## CRediT authorship contribution statement

**Guilherme F. Paganoti:** Writing – review & editing, Writing – original draft, Formal analysis, Data curation, Investigation, Conceptualization, Methodology. **Uenis Tannuri:** Writing – review & editing, Methodology. **Josiane O. Gonçalves:** Writing – review & editing, Methodology. **Suellen Serafini:** Writing – review & editing, Methodology. **Raimundo R.N. Guimarães:** Writing – review & editing, Methodology. **Alessandro R. Belon:** Supervision, Methodology. **Ana Cristina A. Tannuri:** Funding acquisition, Supervision, Writing – review & editing.

## Declaration of competing interest

The authors declare no conflicts of interest.
